# Causal relationship between particulate matter 2.5 and diabetes: two sample Mendelian randomization

**DOI:** 10.3389/fpubh.2023.1164647

**Published:** 2023-08-10

**Authors:** Joyce Mary Kim, Eunji Kim, Do Kyeong Song, Yi-Jun Kim, Ji Hyen Lee, Eunhee Ha

**Affiliations:** ^1^Graduate Program in System Health Science and Engineering, College of Medicine, Ewha Womans University, Seoul, Republic of Korea; ^2^Department of Environmental Medicine, School of Medicine, Ewha Womans University, Seoul, Republic of Korea; ^3^Department of Internal Medicine, School of Medicine, Ewha Womans University, Seoul, Republic of Korea; ^4^Institute of Ewha-SCL for Environmental Health (IESEH), College of Medicine, Ewha Womans University, Seoul, Republic of Korea; ^5^Department of Pediatrics, College of Medicine, Ewha Womans University, Seoul, Republic of Korea; ^6^Department of Medical Science, College of Medicine, Ewha Medical Research Institute, Ewha Womans University, Seoul, Republic of Korea

**Keywords:** particulate matter 2.5, diabetes, genetics epidemiology, environmental epidemiology, two sample Mendelian randomization, GWAS

## Abstract

**Backgrounds:**

Many studies have shown particulate matter has emerged as one of the major environmental risk factors for diabetes; however, studies on the causal relationship between particulate matter 2.5 (PM_2.5_) and diabetes based on genetic approaches are scarce. The study estimated the causal relationship between diabetes and PM_2.5_ using two sample mendelian randomization (TSMR).

**Methods:**

We collected genetic data from European ancestry publicly available genome wide association studies (GWAS) summary data through the MR-BASE repository. The IEU GWAS information output PM_2.5_ from the Single nucleotide polymorphisms (SNPs) GWAS pipeline using pheasant-derived variables (Consortium = MRC-IEU, sample size: 423,796). The annual relationship of PM_2.5_ (2010) were modeled for each address using a Land Use Regression model developed as part of the European Study of Cohorts for Air Pollution Effects. Diabetes GWAS information (Consortium = MRC-IEU, sample size: 461,578) were used, and the genetic variants were used as the instrumental variables (IVs). We performed three representative Mendelian Randomization (MR) methods: Inverse Variance Weighted regression (IVW), Egger, and weighted median for causal relationship using genetic variants. Furthermore, we used a novel method called MR Mixture to identify outlier SNPs.

**Results:**

From the IVW method, we revealed the causal relationship between PM_2.5_ and diabetes (Odds ratio [OR]: 1.041, 95% CI: 1.008–1.076, *P* = 0.016), and the finding was substantiated by the absence of any directional horizontal pleiotropy through MR-Egger regression (β = 0.016, *P* = 0.687). From the IVW fixed-effect method (i.e., one of the MR machine learning mixture methods), we excluded outlier SNP (rs1537371) and showed the best predictive model (AUC = 0.72) with a causal relationship between PM_2.5_ and diabetes (OR: 1.028, 95% CI: 1.006–1.049, *P* = 0.012).

**Conclusion:**

We identified the hypothesis that there is a causal relationship between PM_2.5_ and diabetes in the European population, using MR methods.

## Introduction

Diabetes is a multifactorial disease caused by an interaction of genetic and environmental components (1). Over the past decade there has been a marked increase in its prevalence worldwide and is thus becoming an increasing public health threat (2). Diabetes imposes substantial financial and societal costs upon healthcare systems and society at large (3).

Diabetes's cause is multifactorial and includes genetic and environmental components (1). Some well-established risk factors for diabetes are obesity, sedentary lifestyle habits, an unhealthy diet, family history and increasing age; in addition to these well-recognized risk factors, research is currently investigating any correlation between exposure to ambient air pollution and the development of diabetes (1).

Particulate matter 2.5 (PM_2.5_), an air pollutant that has recently drawn significant media attention for its harmful impacts on respiratory and cardiovascular health, has attracted considerable public attention in recent years (4). PM_2.5_ refers to fine particles with diameters < 2.5 micrometers that can penetrate deeply into respiratory systems and enter bloodstream. Sources that emit this pollution include vehicle exhaust emissions, industrial emissions, combustion processes, etc.

Previous studies have investigated the association between PM_2.5_ exposure and diabetes, and its related complications, and intriguing results. Epidemiological studies conducted across various populations have pointed to an association between long-term PM2.5 exposure and an increased risk of diabetes development—often through measures such as air pollution exposure assessments, biomarker analyses and health outcome evaluations (5, 6).

The exact mechanisms connecting PM_2.5_ exposure with diabetes remain to be understood; it has been hypothesized that it may induce systemic inflammation, oxidative stress and endothelial dysfunction which all play key roles in its pathogenesis (7). Furthermore, exposure has been linked to insulin resistance, impaired glucose metabolism and changes in pancreatic beta-cell function; all of which are fundamental aspects of diabetes development (8).

According to previous studies, genetic polymorphism is an important factor to consider when studying the effects of pollutants on different physiological and immunological functions in humans. For instance, a genetics-based study revealed that women with GPX4-rs376102 AC/CC genotype are more susceptible to air pollutants (9). Epidemiological studies conducted in various populations have indicated a positive correlation between long-term PM_2.5_ exposure and the risk of developing diabetes (5, 6). These studies have employed methods such as assessing air pollution exposure, analyzing biomarkers, and conducting detailed evaluations of health outcomes.

Regarding PM_2.5_ and diabetes, MR studies employ genetic variants associated with exposure as instrumental variables. Unaffected by confounders or reverse causation effects, they allow researchers to estimate the causal impact of PM_2.5_ exposure on diabetes risk estimation. By using large-scale genetic data sets along with robust statistical techniques, these MR studies may offer valuable insight into any possible causal relationships between PM_2.5_ exposure and diabetes risk.

Mendelian Randomization (MR), which utilizes genetic variations as instrumental variables and SNP data from GWAS to explore causal relationships, was applied to investigate PM_2.5_'s possible link to diabetes (10). This approach reduces biases and has profound implications for public health interventions and preventive strategies. First, using genetic variants as instrumental variables makes the method more reliable to examine causation than traditional observational research methods; thus, minimizing bias. Second, establishing the link between PM_2.5_ and diabetes through multivariate analysis could have serious ramifications for public health interventions and preventive measures designed to lower air pollution and lessen diabetes risks among the general population. Therefore, we created an alternative hypothesis suggesting a causal relationship between PM_2.5_ and diabetes and performed two-sample MR analysis to accept/reject it using data available through GWAS (11, 12).

## Methods

### Study population and data sources

The genetic data for this study were retrieved from GWAS summary data. The data is available in the MR-BASE repository. The repository was created by the Medical Research Council Integrative Epidemiology Unit, University of Bristol, for facilitating TSMR created the repository made repository. The GWAS outcomes depicted are insufficiently precise, which destabilize the effective application of this analysis (12). The referred repository (http://www.mrbase.org) comprises 11 billion SNP-trait associations from 1,673 GWAS. The repository is updated regularly (11).

The MRC-IEU UK Biobank genome wide association study (GWAS) pipeline has been optimized to conduct GWAS quickly, effectively, and uniformly on the imputed genetic dataset of the full 500,000 from UK Biobank. Participants were aged between 40 and 69 years when they joined UK Biobank between 2006 and 2010. Each participant attended a baseline assessment at a center in England (89%), Scotland (7%) and Wales (4%) (13).

The IEU GWAS information output PM_2.5_ from the SNPs GWAS pipeline using pheasant derived variables consortium MRC-IEU. The annual relationship of PM_2.5_ (2010) were modeled for each address using a Land Use Regression model developed as part of the European Study of Cohorts for Air Pollution Effects. For the outcome variable diabetes, the GWAS data were obtained from the MRC-IEU. Diabetes GWAS information consortium MRC-IEU were used, and the genetic variants were used as the IVs.

The PM_2.5_ GWAS summary dataset (GWAS ID: ukb-b-10817) included 423,796 participants of European ancestry. PM_2.5_ concentrations at participants' home addresses were estimated using a Land Use Regression (LUR) model (14). The diabetes was diagnosed by doctor (output from GWAS pipeline using pheasant derived variables from UK Biobank) GWAS summary dataset (GWAS ID: ukb-b-10753) contained 461,578 individuals of European descent, including 22,340 cases and 439,238 controls.

The number of European participants with a PM_2.5_ phenotype was 423,796, while that for diabetes in the same population was 461,578, suggesting predisposition of PM_2.5_ phenotype to diabetes. The GWAS data was retrieved from the MR-BASE repository.

**Figure 1:**
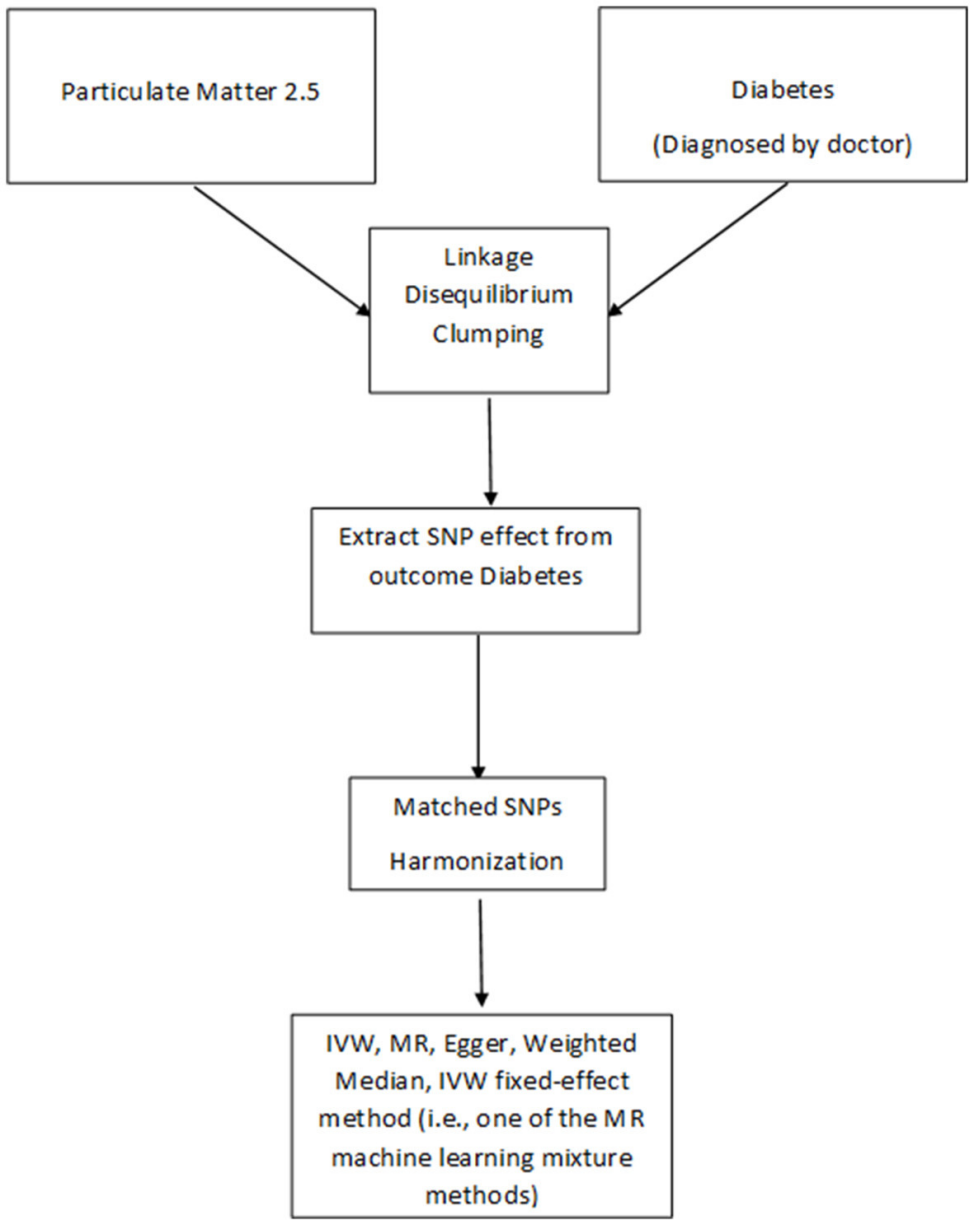
Flow chart of Mendelian randomization.

The total 9,851,867 PM_2.5_ SNPs were using a Bonferroni statistical threshold (*p* < 5 × 10 – 8). Linkage disequilibrium (LD) was used to identify the independent SNPs by using the R^2^ threshold < 0.005. After adjusting for correlated SNPs, 7 of them were selected as the genetic instruments for evaluating genetic predisposition to being PM_2.5._ Once the genetic instruments were selected, the final set of harmonized data were completed by extracting information from the outcome GWAS matched to each instrument SNP (Figure 1).

### Assumptions of mendelian randomization

**Figure 2:**
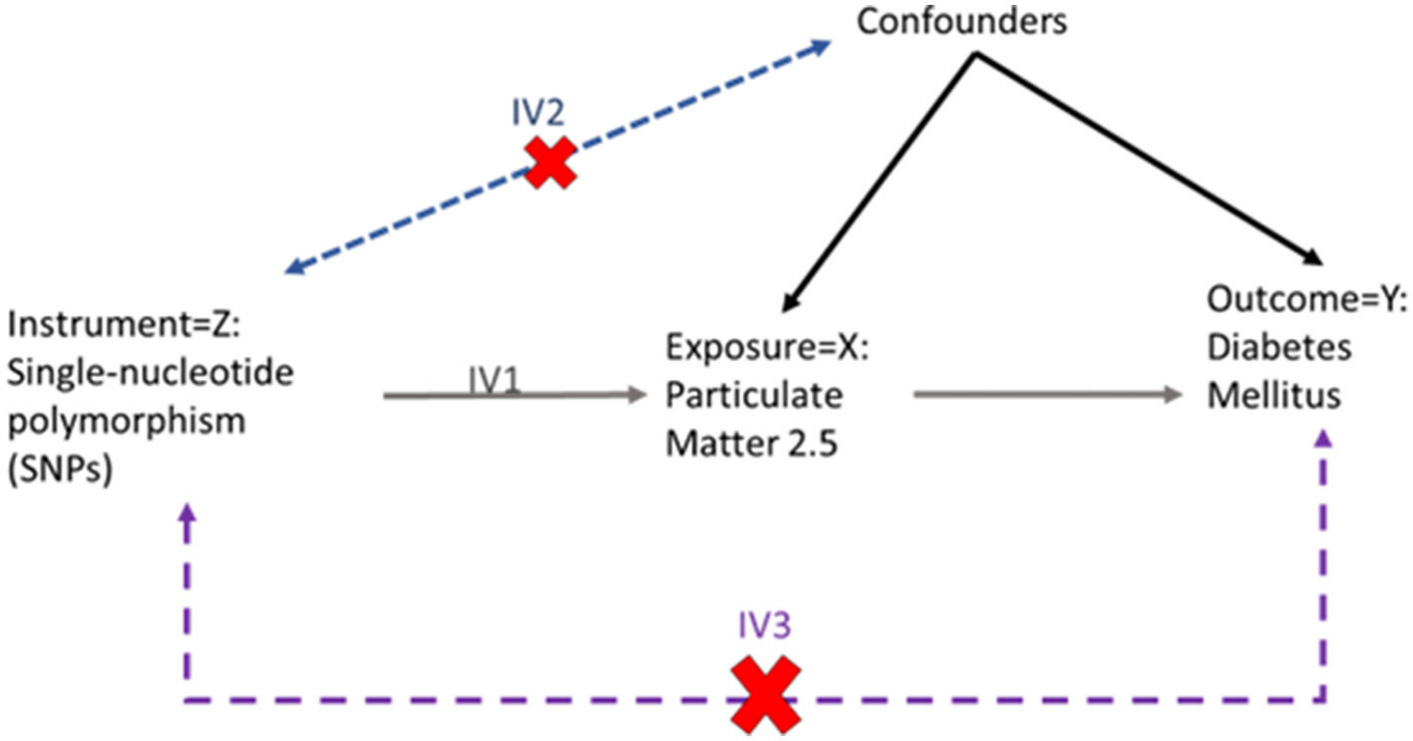
Flow diagram of two-sample Mendelian randomization.

In this Study, the TSMR approached was used. Where causal relationship between PM_2.5_ and diabetes are obtained by dividing the instrument outcome associated by the instrument exposure association of each SNP (Figure 2). These association ratios are then combined using the IVW method for the main MR analysis (15).

MR estimate the valid, the instruments must satisfy three key assumptions (Figure 2):

IV1. The instruments must be robustly associated with the exposure.
IV2. The instruments must not be associated with any confounders of the exposure-outcome relationship.
IV3. The instruments can only be associated with outcome via the exposure and not via a different biological pathway independent of the exposure.

### Statistical analysis

To evaluate the causal directionality between PM_2.5_ and diabetes, we performed three representative MR methods: IVW, Egger, and Weighted median for causal relationship using genetic variants (16, 17). Furthermore, we applied a mixture-of-experts machine learning framework (MR Mixture) to improve the performance of MR estimation after identifying outlier SNPs. Since the assumption of MR can be violated due to SNPs that have horizontal pleiotropy, there were various attempts to develop methods lowering level of horizontal pleiotropy (18). Machine learning algorithms can help identify potential pleiotropic variants (genetic variants that affect multiple traits), and assess their impact on multivariate analysis. Furthermore, machine learning approaches may assist in creating genetic risk scores or polygenic risk scores to capture the combined effects of multiple genetic variants. Machine Learning methods commonly utilized in MR include regularized regression and mixture-of-experts machine learning framework (MR Mixture). They can help with variable selection, prediction modeling and exploring complex relationships among genetic instruments, exposures and outcomes (19). The MR Mixture is one of automatic model selection based on Random Forest algorithm to select the most appropriate method across a range of different MR strategies. MR strategies contains the combination of two instrument selections (Top hits, Steiger filtering) to identify outlier and fourteen MR estimation method (IVW fixed effects meta-analysis, IVW random effects meta-analysis, Egger fixed effects, Egger random effects, Rucker point estimate, Rucker mean of the jackknife, Rucker median of the jackknife, simple median, weighted median, penalized weighted median, simple mode, weighted mode, each weighted with or without the assumption of no measurement error in the exposure estimates) (20).

The fixed effects meta-analysis assumes that the only source of differences between relationship across the studies is due to sampling variation. In the MR context this translates to each SNP exhibiting no horizontal pleiotropy. Gene must be valid instruments. If all SNPs exhibit horizontal pleiotropy, then the effect estimate is asymptotically unbiased, but the standard error will be overly precise. Uses weights that assume the SNP-exposure association is known, rather than estimated, with no measurement error (i.e., known as the NOME assumption). Causal effect relationship from the IVW approach exhibit weak instrument bias whenever SNPs used as IVs violate the NOME assumption, which can be measured using the F-statistic with IVW methods (21, 22).

The leave-one-out sensitivity method was performed to compute whether random relationship were affected by an individual genetic locus. For further interpretation, scatterplots, forest plots, and funnel plots were also produced (11).

In this study, all MR analyses were calculated using R packages in R version 4.1.1 from the R Core Team, based in Vienna, Austria.

## Results

The inclusion of sample sizes in Table 1 indicates the number of individuals from whose genetic data and pertinent information were acquired for each dataset. These datasets are critical for undertaking Mendelian randomization (MR) analyses, which use genetic variants linked with an exposure (in this case, PM_2.5_) to assess the causal impact on an outcome of interest (in this case, diabetes, and its associated risk factors). For genetic instruments, PM_2.5_, and diabetes GWAS data were obtained for European ancestry.

**Table 1:** Description of GWAS consortium used for exposure and outcome.

**Variable**	**Phenotype**	**Population**	**Sex**	**Sample size (cases)**	**Unit**	**Consortium**
Exposure	Particulate Matter (PM_2.5_)^b^	European	All	423,796	SD	MRC-IEU^a^
Outcome	Diabetes diagnosed by doctor	European	All	461,578 (22,340)	Log odds	MRC-IEU^a^

a MRC-IEU, MRC integrative epidemiology unit.

b UK BIOBNANK ID 24006: Output from GWAS pipeline using Pheasant derived variables from UK Biobank Consortium.

^*^Standard deviation.

The fact that the GWAS data were obtained for people of European ancestry implies that the findings and genetic tools found in this investigation are most applicable and relevant to people of European heritage. Because genetic architecture and allele frequencies change between populations, it is critical to consider ancestry while conducting MR analysis or interpreting the results.

The Table 2 shows the IVW method, we revealed the causal relationship between PM_2.5_ and diabetes (Odds ratio [OR]: 1.041, 95% CI: 1.008–1.076, *P* = 0.016), and the finding was substantiated by the absence of any directional horizontal pleiotropy through MR-Egger regression (β = 0.016, *P* = 0.687). From the IVW fixed-effect method (i.e., one of the MR Mixture methods), we excluded outlier SNP (rs1537371) and showed the best predictive model (AUC = 0.72) with a causal relationship between PM_2.5_ and diabetes (OR: 1.028, 95% CI: 1.006–1.049, *P* = 0.012).

**Table 2:** Causal relationship between particulate matter (PM_2.5_) and diabetes.

**Exposure**	**Outcome**	**Method**	**Nsnp** ^a^	β	**SE** ^b^	**OR (95% CI)** ^c^	***P***
Particulate Matter (PM_2.5_)	Diabetes (diagnosed by doctor)	IVW^d^	7	0.040	0.017	1.041 (1.008–1.076)	0.016
		MR Egger	7	0.016	0.057	1.017 (0.906–1.140)	0.791
		Weighted median	7	0.034	0.017	1.035 (1.001–1.070)	0.040
		Fixed-Effect IVW	6	0.027	0.01	1.028 (1.006–1.049)	0.011

a Nsnp, number of (single nucleotide polymorphism, SNP).

b SE, standard error.

c OR, odd ratio (95% confidence intervals).

d IVW, inverse variance weight.

**Figure 3:**
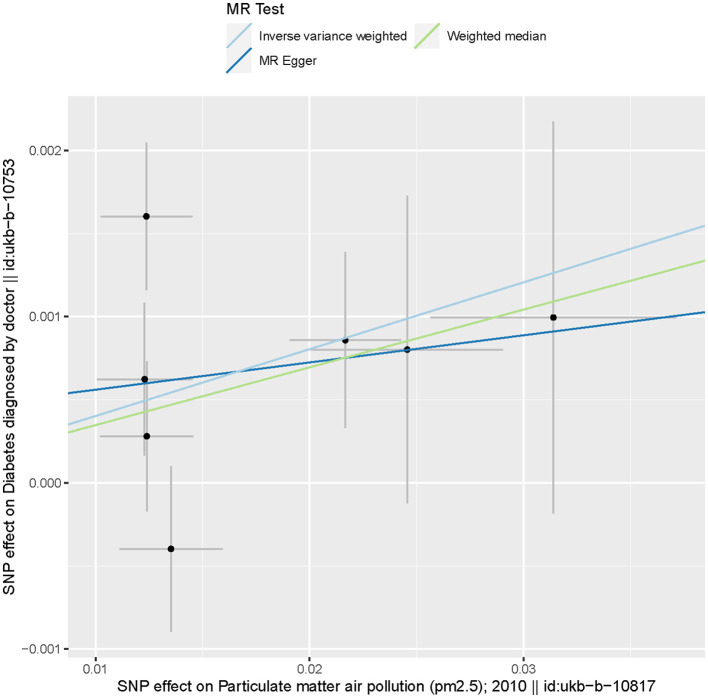
Scatter plots of genetic associations with particulate matter 2.5 against the genetic associations with diabetes. The slopes of each line represent the causal association for each method. The blue line represents the IVW estimate, the green line represents the weighted median estimate, and the dark blue line represents the MR-Egger estimate.

Based on the two-sample MR randomization, it was revealed that a causal association between PM_2.5_ and diabetes existed. The MR slopes of the plots for the IVW and weighted median regression indicated positive direction plots and were statistically significant suggesting a causal relationship between the measurement variable SNP PM_2.5_ on diabetes (Figure 3). On the contrary, the MR-Egger regression indicated that the slope (causal effect) had no significant relationship with the outcome. This assumption suggests that there might be horizontal pleiotropy or significant outliers that violate the findings of the IVW, and weighted mean regarding the relationship between genetic predispositions to PM_2.5_ and diabetes. The Figure 3 substantiated the assumption as the effect size on SNP-based outcome was lowest for MR-Egger.

**Figure 4:**
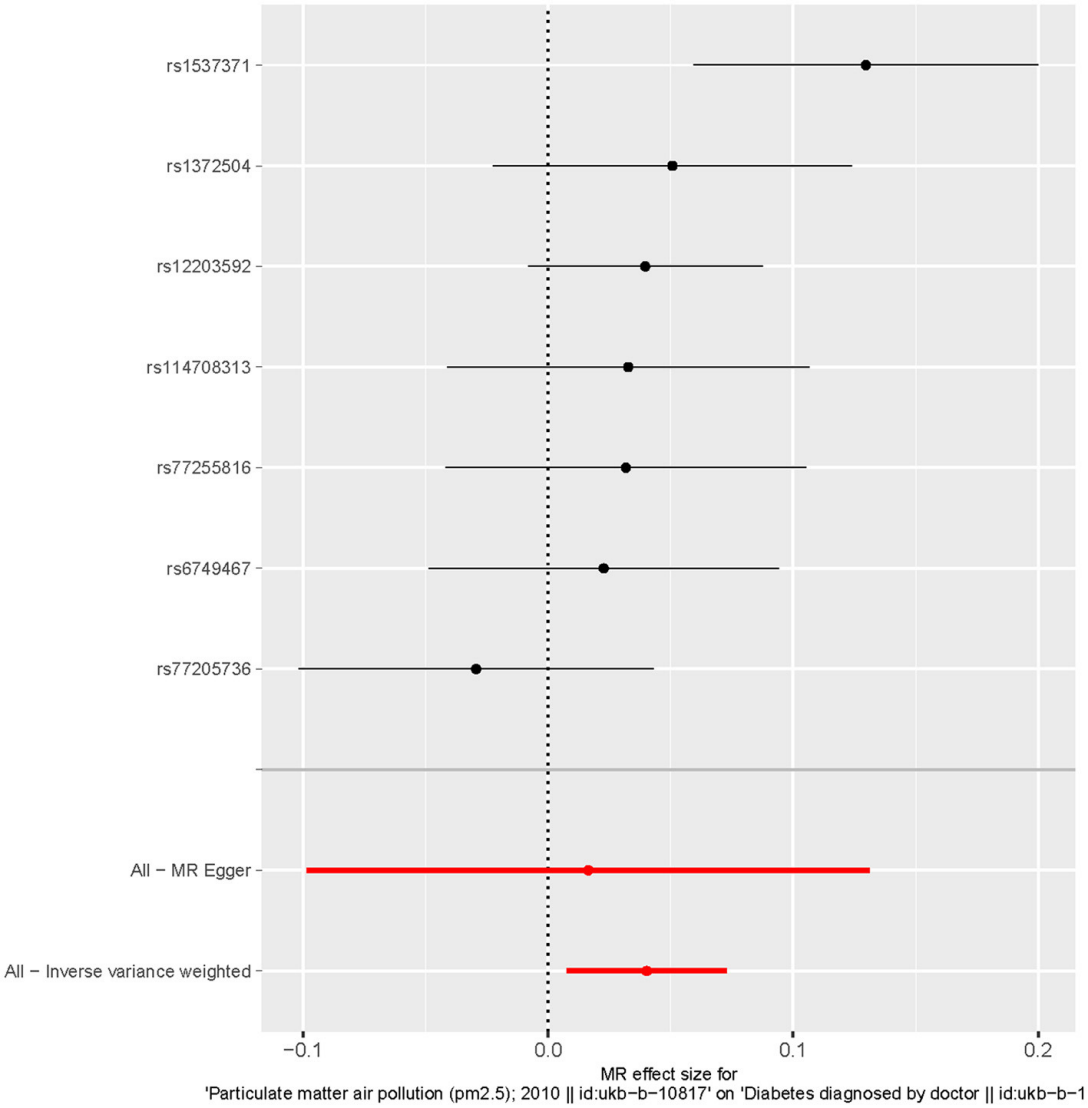
Forest plot of the causal effects of particulate matter 2.5 associated SNPs on diabetes.

**Figure 5:**
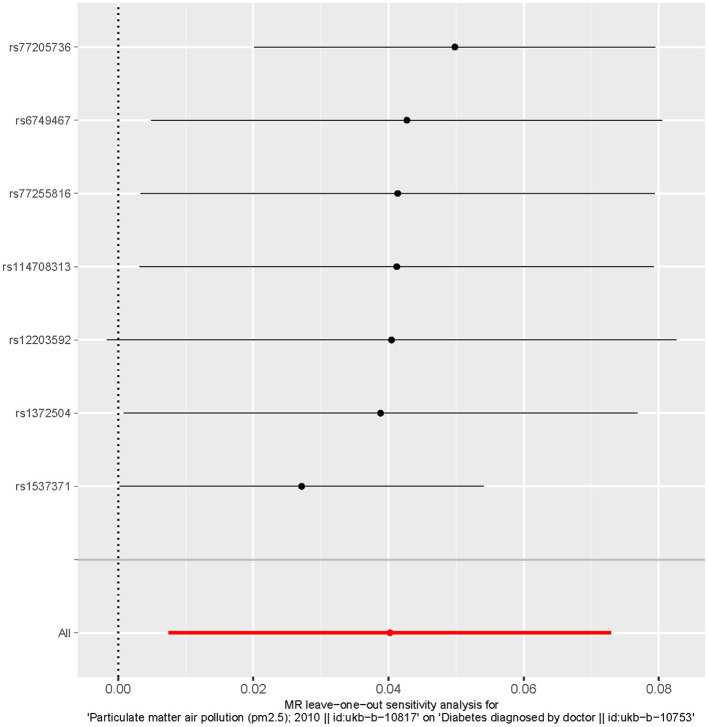
Leave one out the sensitivity analysis plot-the causal effect of particulate matter 2.5 on diabetes.

**Figure 6:**
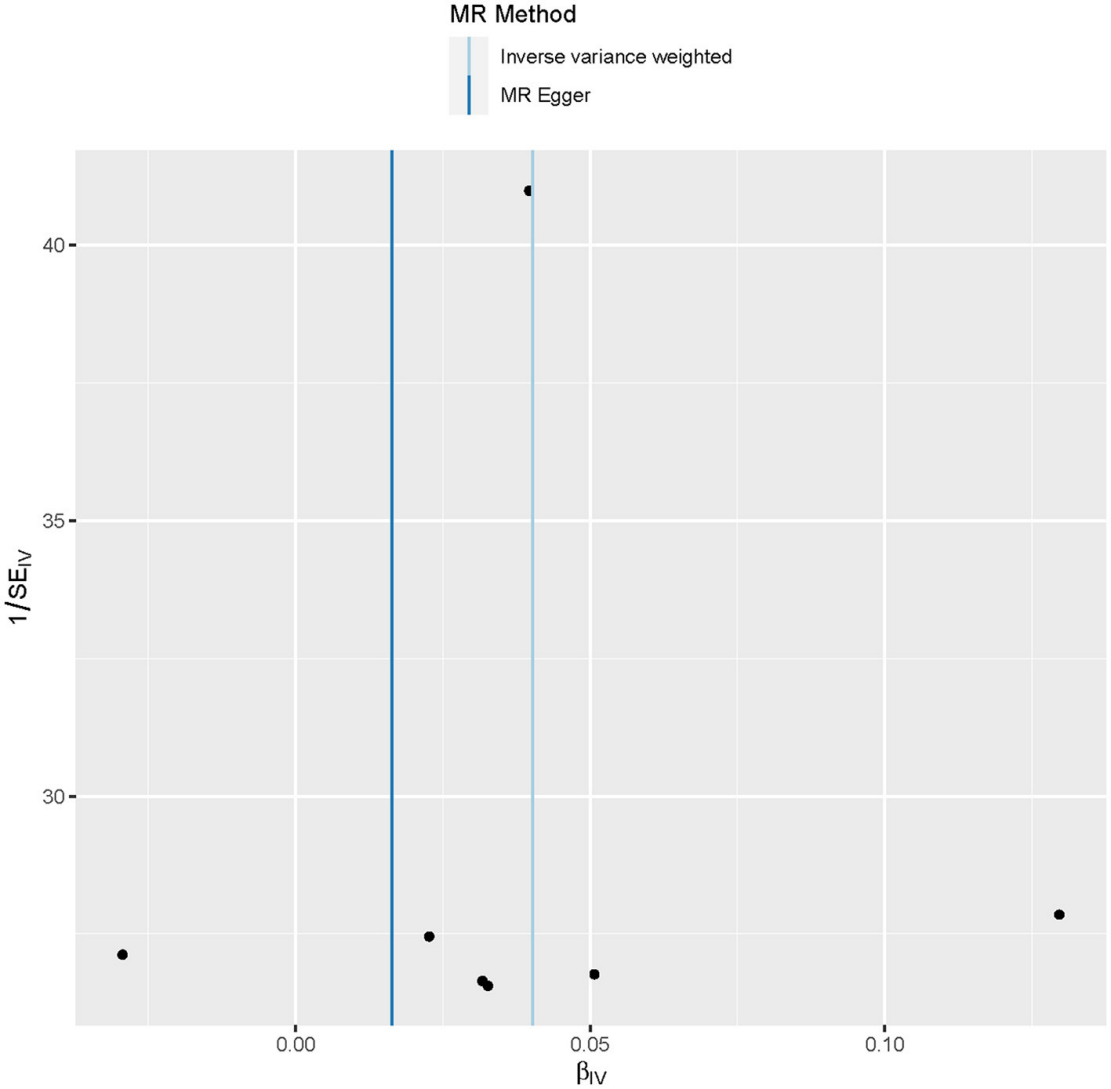
Funnel plot showing the relationship between the cause-effect of particulate matter 2.5 and diabetes.

MR analysis identified an outlier with the estimated causal effects suggested having been >2 SDs off the average causal effect that was obtained from the 7 PM_2.5_ SNPs. Furthermore, the MR size had an effect on the PM_2.5_'s association with diabetes based on the all IVW and all MR-Egger relationship, indicating varied MR effects for these two measurements (Figure 4). However, all the measurements involving both MR-Egger and IVW relationship for MR-leave-one-out revealed a stronger association between PM_2.5_'s and diabetes. Such assumptions were ruled out when one of the SNPs were left out from the analysis (Figure 5). The respective SNP could be considered a potential outlier for the relationship between PM_2.5_ and diabetes. On the other hand, the funnel plot (Figure 6) showed that MR-Egger produced relatively more asymmetry for the effects of PM_2.5_ on diabetes compared to IVW.

## Discussion

This study explored the causal relationship between PM_2.5_ and diabetes as a function of TSMR using suitable platforms. The MR-Egger estimate reflected the absence of horizontal pleiotropy on diabetes as its intercept was positive but non-significant (β = 0.016, *P* = 0.687). Genetically predisposed PM_2.5_ was significantly related to the increased risk of diabetes as depicted by the IVW estimation (Odds ratio [OR]: 1.041, 95% CI: 1.008–1.076, *P* = 0.016).

MR is gaining high popularity in epidemiological studies because it helps to establish whether a modifiable exposure has a causal relationship with the pathophysiology of a disease (16). Also, MR is increasingly used due to the availability of GWAS that provides an opportunity to use a large number of genetic variants in the referred analysis. If the variants in totality could explain a larger proportion of variance in the exposure variable, it would lead to more precise relationship of the causal effects. The precise estimation would increase the reliability of the cause-effect relationships with the referred variables. On the contrary, analysis conducted with an enlarged set of genetic variants is more likely to incorporate invalid instrument variables due to the violations of the assumptions of MR. One such set of variants are those causing horizontal pleiotropy. This is in contrast to vertical pleiotropy, where two traits that are biologically related are correlated irrespective of the gene or variant that is responsible for the effect. The study explored whether genetically predisposed PM_2.5_ significantly increases the risk of diabetes.

PM_2.5_ acts as a mediator linking endothelial dysfunction and insulin resistance. Alterations in endothelial function have been implicated in reduced peripheral glucose uptake (18). In addition, tumor necrosis factor (TNF-a), interleukin-6 (IL-6), resist in, and leptin levels were elevated with PM_2.5_ exposure, in keeping with a proinflammatory insulin-resistant state.

In addition, PM exposure results in elevations in prothrombotic adipokines such as plasminogen activator inhibitor 1 and increased circulation adhesion molecules such as intracellular adhesion molecule-1 and E-selectin.

Sun et al. also reported experimental evidence of mouse model (23). PM_2.5_ exposure exaggerates insulin resistance and visceral inflammation and adiposity, so these findings proved a new link between air pollution and type 2 DM. PM exposure was associated with impairment in phosphatidylinositol 3-kinase–Akt–endothelial nitric oxide synthase signaling in the aorta and decreased tyrosine phosphorylation of IRS-1 in the liver, providing evidence for abnormal insulin signaling in the vasculature. In addition, Liu et al., also suggested that PM_2.5_ -mediated alterations in glucose homeostasis and PM_2.5_-mediated inflammation in visceral adipose tissue (24). Toll-like receptors (TLRs) and nucleotide oligomerization domain receptors (NLRs) can be mediated as particular matter sensors. Long et al. studied systemic increase in IL-6 may play an important role in the deterioration of the type 2 DM via IL-6/signal transducer and activator of transcription 3 (STAT3)/suppressor of cytokine signaling (SOCS) pathway in liver after short-term exposure to PM_2.5_ (25).

Evidence from epidemiological studies, combined with animal and toxicologic experiments, supports the notion that inflammatory responses to environmental factors are the key mechanism that helps explain the emerging epidemic in metabolic diseases like diabetes. Both genetic and environmental factors undoubtedly play a role, in the emergence of such diseases but the contributions of the physical and social environment determining susceptibility may also be critical. Nontraditional factors such as air pollution that are pervasive in the urban environment may together with other dominant factors provide synergism in accelerating the propensity for T2DM.

Future studies are warranted to gain greater insight into the molecular mechanisms involved (e.g., intermediary, and intracellular signaling pathways), the responsible pollutants (e.g., components, sizes/sources), the role of combined exposures to mixtures (e.g., ozone plus PM), and susceptibility factors (e.g., gene-environment interactions, vulnerable populations).

The purpose of a machine-learning application is different by its field or data but mainly could be used for improving performance of a predictive model. However, despite the popular use of machine learning, prior studies show that applying machine learning to GWAS is still rare. In the current study, MR mixture based on machine-learning algorithm showed its potential for making higher performance in estimation and lowering bias in estimates. This is because it was possible not only to improve the prediction of causal estimates but also to select important instrumental variables through automatic data-driven methods. In addition, MR mixture turned out to predict unbiased causal estimates with higher power compared to existing traditional methods (20). Although further evidence needs to be accumulated in future, we could suggest that machine-learning applications for GWAS (e.g., MR mixture) had quite a degree of feasibility and efficiency.

## Strengths and limitations

The main strength of this study is large-scale GWAS data was used for the MR analysis. The large sample size allowed for reliable causal effect estimation, assessing the consistency of associations across different MR methods. The MR-Egger approach reduced the bias due to reverse causality and confounding. The IVW coupled with MR-Egger increased the reliability and reproducibility of the study across different comorbid conditions related to diabetes. Second, we robustly confirmed the causal relationship between PM_2.5_ and diabetes through traditional MR estimation and machine-learning MR estimation. Traditional methods alone cannot thoroughly exclude the potential possibility of violating the horizontal pleiotropy assumption. It takes a lot of effort to find the best method among several MR strategies that had the most predictive and lower level of horizontal pleiotropy for the data. The data-driven MR-Mixture method can automatically find the best combination of the outlier filtering method and the MR estimation method based on the machine-learning method. Both traditional and MR-mixture methods showed a prominent causal relationship between PM_2.5_ and diabetes, and we found that the estimation of causal effects could be improved when outliers were removed from traditional methods based on the MR-Mixture method.

The major limitation of this study is that we only used the data from individuals of European descent. Therefore, it should be cautious about generalizing our findings to other populations. Another limitation of this study was the small sample size that might have increased the risk of Type-I error. Also even if diabetes was diagnosed by a doctor in UKB platform, it is not well known to determine what the diagnostic criteria were type 1 diabetes or type 2 diabetes.

Moreover, no power estimations were conducted for selecting the sample size, which might have further reduced the reproducibility of the findings. Nevertheless, the limited availability of population-specific information on genetic associations, genetic instruments tend to show poor statistical power. On the contrary, different MR frameworks substantiated the causal relationship between the genetic predisposition of being PM_2.5_ and diabetes after removing the outliers. Such measures increased the reliability and validity of the findings of our study.

## Conclusion

We identified the hypothesis that there is a causal relationship between PM_2.5_ and diabetes in the European population, using MR methods. Therefore, the findings from this study discovered that person exposed to more PM_2.5_ was strongly related to higher risk of diabetes in European population.

## Data availability statement

Publicly available datasets were analyzed in this study. This data can be found here: https://www.mrbase.org/.

## Author contributions

JK: conceptualization, methodology, investigation, formal analysis, data curation, and writing—original draft. EK: conceptualization, methodology, and writing—original draft. DS: introduction, methodology, and writing—original draft. Y-JK and JL: methodology and writing—original draft. EH: conceptualization, methodology, writing—original draft, and supervision. All authors contributed to the article and approved the submitted version.
